# Knowledge, Attitudes, and Practices of Oral Care for Residential Older People Among Healthcare Providers in Macao

**DOI:** 10.3390/geriatrics11030058

**Published:** 2026-05-13

**Authors:** Miffy M. F. Lam, Florence M. F. Wong

**Affiliations:** 1 Estrada Conde de S. Januário, Conde de S. Januário General Hospital, Macao 999078, China; lmf2930@gmail.com; 2 School of Nursing, Tung Wah College, Hong Kong SAR, China

**Keywords:** oral care, knowledge, attitudes, practices, healthcare providers, older residents, long-term care, nursing homes

## Abstract

**Background/Objectives**: Oral health is integral to the overall well-being and quality of life of older adults. For institutionalized older residents who depend on staff for daily care, the knowledge, attitudes, and practices (KAP) of healthcare providers directly influence oral health outcomes. This study aimed to assess oral care KAP among healthcare providers in Macao’s residential care facilities and to examine the relationships between KAP and personal characteristics. **Methods**: A cross-sectional study was conducted from September to December 2023 using a structured KAP questionnaire. Data were collected from 225 healthcare providers across four nursing homes in Macao. **Results**: Significant positive correlations were found among KAP (Spearman’s γ = 0.293–0.419; *p* < 0.001). Mean scores were 15.60 (SD = 2.27)/19 for knowledge, 52.13 (SD = 5.75)/65 for attitudes, and 45.87 (SD = 5.93)/55 for practices. Denture care was the major knowledge deficit. Knowledge was associated with age 18–20 (*p* = 0.002), age 31–40 (*p* = 0.015), and university education (*p* = 0.003). Attitudes were associated with age 18–20 (*p* = 0.002), female gender (*p* = 0.005), and primary education (*p* = 0.044). Practice was associated with training programme attendance (*p* = 0.006) and female gender (*p* = 0.016). All associations involving the age 18–20 subgroup (*n* = 3) and male gender (*n* = 16) should be interpreted with caution due to very small sample sizes. **Conclusions**: Oral care KAP among HAs and PCWs in Macao’s residential care homes are positively but modestly correlated. Most providers acquired knowledge through induction training with verified competency outcomes and personal experience. These findings highlight the need for structured, repeatable training and suggest that Macao’s data can contribute to international efforts to improve oral care in institutionalized aging populations. Future research should employ longitudinal or observational designs and include all elderly care facilities in Macao.

## 1. Introduction

Macao is undergoing rapid population aging; individuals aged 65 and over now constitute 14.6% of the population, officially designating it an “aging society” with a projected transition to an “ultra-aged society” by 2036 [1]. An ‘aging society’ is defined as a population where 7–14% of individuals are aged 65 years or older, while an ‘ultra-aged society’ refers to a population where this proportion exceeds 21% [2]. This demographic shift drives increasing demand for long-term care and rising healthcare expenditures [3], making preventive health promotion a strategic priority.

Oral health is a critical yet often neglected component of overall well-being and quality of life in older adults [4,5]. Oral diseases are highly prevalent in this population; for example, a large proportion of nursing home residents have untreated caries and periodontal disease [5]. Moreover, poor oral hygiene is a significant risk factor for serious systemic conditions, including aspiration pneumonia, cardiovascular disease, poor glycemic control, and malnutrition, contributing to higher morbidity and mortality [6,7,8].

For the growing number of older adults in long-term care, daily oral hygiene depends largely on non-dental staff, particularly health assistants (HAs) and personal care workers (PCWs). These frontline providers face well-documented barriers, including lack of formal training, task aversion, high workloads, and resident resistance, especially among those with cognitive impairments [9,10,11]. As a result, oral care is often deprioritized or performed inadequately [10,11].

The knowledge, attitude, and practice (KAP) model offers a useful framework to understand these challenges [12,13]. Healthcare providers’ KAP are interlinked and influence oral health outcomes [14]. However, existing international studies have primarily focused on registered nurses in acute care settings [15,16,17], with limited attention to HAs and PCWs who deliver most daily oral care in long-term facilities. Furthermore, few studies have applied Capability, Opportunity, Motivation, and Behaviour (COM-B) model [18] to examine how capability, opportunity, and motivation interact to influence oral care behaviors in residential settings.

Macao presents a unique yet internationally relevant context. As a Special Administrative Region of China with a highly integrated Eastern and Western healthcare system, rapid population aging (projected to reach “ultra-aged” status by 2036), and no established outreach dental services for nursing homes, findings from Macao can inform similar rapidly aging societies in East Asia and beyond where oral health infrastructure may lag behind demographic shifts. Despite the importance of this context, how personal characteristics (e.g., job role, training history) relate to oral care KAP among Macao’s healthcare providers remains unclear.

To address this gap, the present study assesses the current state of oral care KAP among healthcare providers in Macao’s elderly care facilities and examines the relationship between KAP and key demographic and occupational factors. The study aims to provide evidence-based insights into developing targeted training and support strategies to improve oral health and quality of life for Macao’s institutionalized older adults.

## 2. Materials and Methods

### 2.1. Design

This study employed a cross-sectional, quantitative design to investigate the KAP of healthcare providers regarding oral care for older residents in residential care facilities. To ensure methodological rigor and transparency, the research followed the Strengthening the Reporting of Observational Studies in Epidemiology (STROBE) guidelines [19] (See S1 in Supplementary Materials).

### 2.2. Theoretical Framework

**Figure 1:**
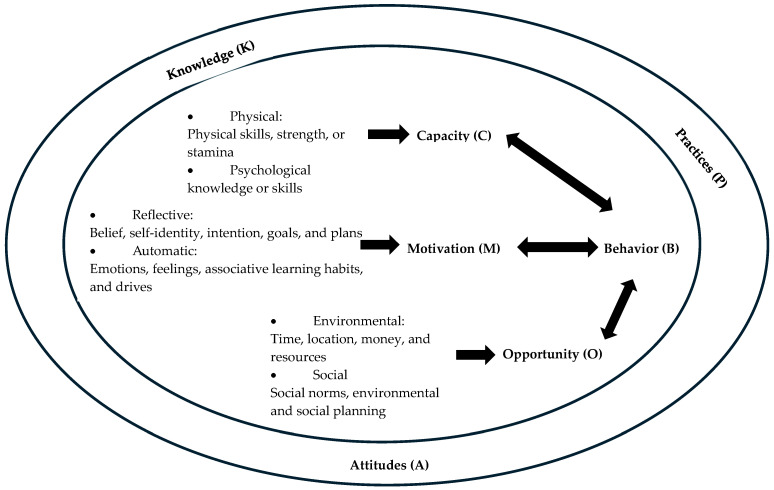
The COM-B Model.

Guided by Michie et al.’s COM-B model [16], this study adopts its core premise: for a behaviour (B) to occur, an individual must have the necessary capability, opportunity, and motivation. As illustrated in Figure 1, this study operationalizes the model as follows:

Capability: Knowledge of oral health and care procedures;
Opportunity: External factors such as resources, time, and workplace culture;
Motivation: Attitudes towards providing oral care;
Behaviour: The outcome, defined as self-reported oral care delivery practices.

The model also acknowledges that opportunity directly influences both the behaviour and the other components.

### 2.3. Participants and Settings

This study population comprised HAs and PCWs employed in older residential care facilities in Macao, as these roles are directly responsible for providing daily oral care to residents.

The inclusion criteria for participants were as follows: (1) aged 18 years or older; (2) currently employed as care staff providing oral care services for older residents at an older residential care facility; and (3) proficient in Chinese to understand the survey. The sample size was determined a priori using G*Power software (Version 3.1.9.7). Based on a similar study by Wong et al. [15] that reported an effect size of R^2^ = 0.31, a linear multiple regression analysis with an alpha (α) of 0.05 and a statistical power (1 − β) of 0.95 required a minimum of 114 participants. To accommodate an estimated 20% attrition rate, the target sample size increased to 137.

The research proposal was distributed via email to all seven eligible nursing homes or elderly integrated service centers in Macao. Four institutions agreed to participate. Ultimately, a total of 225 valid questionnaires were collected, exceeding the initial target and enhancing the statistical power of the analysis.

### 2.4. Instruments

The instruments consisted of two parts: a demographic survey and a standardized KAP questionnaire on oral care.

#### 2.4.1. Demographic Survey

A self-developed section collected participants’ personal and professional background information, including gender, age, years of healthcare experience, educational level, job position, and history of oral care training (including duration and knowledge sources, such as life experience, self-study, peer teaching, onboard training, professional course content, and supervisor guidance).

#### 2.4.2. KAP Survey

The KAP questionnaire on oral care for older residents was developed and validated by Wong et al. [20]. It comprises 43 items: 19 for knowledge, 13 for attitudes, and 11 for practice.

Knowledge items evaluate foundational understanding of oral care. Items have three response options: “Yes,” “No,” and “Don’t know.” A correct answer scores 1 point, while an incorrect or “Don’t know” response scores 0 points. Total scores range from 0 to 19, with higher scores indicating greater knowledge.

Attitude items assess attitudes and values regarding oral care provision using a 5-point Likert scale (1 = strongly disagree to 5 = strongly agree). The scale includes six reverse-scored items. Total scores range from 13 to 65, with higher scores reflecting more positive attitudes.

Practice items examine the frequency and quality of oral care procedures performed over the past three months, using a 5-point Likert scale (1 = Strongly Disagree to 5 = Strongly Agree). Total scores range from 11 to 55, with higher scores indicating better adherence to recommended practices.

Internal consistency, measured by Cronbach’s alphas, was 0.67 for knowledge, 0.76 for attitudes, 0.85 for practice, and 0.85 for the overall KAP scale. These values indicate acceptable reliability for knowledge and good reliability for attitudes, practice, and the overall scale.

### 2.5. A Pilot Study

To ensure contextual relevance, one item, “Outreach dentists are helpful for our oral care provision to residents”, was slightly modified to “Outreach dentists are helpful for our oral care provision to residents if available,” as no such dental services exist in Macao. A pilot study was then conducted with 15 healthcare providers from long-term care facilities to assess the viability of the modified KAP survey. The internal consistency (Cronbach’s alpha) results were 0.85 for knowledge, 0.75 for attitudes, 0.90 for practices, and 0.90 for the overall KAP.

### 2.6. Ethical Consideration

Ethical approval was granted by the Research Ethics Committee of Macau University of Science and Technology (reference number: MUST-FMD-20230718001). Participation was voluntary, and all eligible participants provided written informed consent prior to data collection. Anonymized serial numbers were assigned to ensure confidentiality, and no identifiable information was linked to responses during analysis. All data were securely stored and kept confidential in accordance with ethical standards for human subject research.

### 2.7. Study Procedure

The study commenced after obtaining ethics approval. In-charge nurses or institutional managers were invited to participate. To minimize disruption to normal institutional operations, the principal investigator (PI) received a list of eligible participants for recruitment and data collection. The PI explained the study details to eligible subjects before requesting them to sign an informed consent form. Subsequently, all enrolled subjects completed a set of questionnaires, including the demographic form and KAP survey, which took approximately 20 min.

### 2.8. Data Analysis

Data were analyzed using SPSS for Windows version 27. Descriptive statistics, including percentages, means, frequency distributions, and standard deviations, were employed to analyze demographic characteristics and outcome variables (KAP). Inferential statistics, including independent samples *t*-tests, bivariate analysis, and one-way ANOVA, were conducted to explore the relationships between demographic variables and KAP outcomes. To identify predictors associated with KAP, a stepwise multivariable regression analysis was performed.

For chi-square analyses, continuous variables (knowledge, attitudes, and practices scores) were categorized into quartiles or clinically meaningful cutoffs as appropriate. Degrees of freedom reflect the number of categories in each analysis after accounting for any cells with expected frequencies <5, which were collapsed where necessary. All *p* values are two-tailed.

## 3. Results

### 3.1. Participants’ Demographic Characteristics

A total of 225 valid questionnaires were collected from four participating elderly care homes. Of the 225 respondents, 154 were PCWs (68.4%) and 71 were HAs (31.6%). Most PCWs were aged 41–50 years, comprising 51.95% of the PCW group, whereas the majority of HAs were aged 21–30 years, accounting for 59.15% of the HA group. A significant gender difference was observed between the two groups (*p* = 0.003). Participants’ background data are presented in Table 1.

The majority of both HAs and PCWs reported receiving oral care knowledge through onboard training (66.23%) and their own experience (64.94%). Notably, a higher proportion of HAs (61.9%) acquired oral care knowledge from their professional training programmes compared with PCWs.

### 3.2. Correlations Among KAP

Bivariate analysis revealed significant positive correlations among all three KAP domains (*p* < 0.001). As shown in Table 2, knowledge was positively correlated with attitudes (γ = 0.34) and practices (γ = 0.35), while attitudes were positively correlated with practices (γ = 0.37). All correlations used Spearman’s rank correlation coefficient due to non-normal distributions.

### 3.3. Associations with Oral Care Knowledge

Chi-square tests were used to examine associations between personal background variables and total knowledge scores (Table 3). Gender was the only variable showing a statistically significant difference in knowledge scores (χ^2^ = 33.27, df = 1, *p* = 0.002).

One-way ANOVA revealed no significant differences in knowledge scores by years of nursing work experience (F(4, 220) = 3.126, *p* = 0.14) or educational level (F(4, 220) = 2.409, *p* = 0.05). However, knowledge scores differed significantly by age group (F(4, 220) = 3.333, *p* = 0.011). Bivariate analysis showed that participants aged 31–40 years had a small but significant negative association with knowledge scores (Spearman’s γ = −0.159, *p* = 0.017). No other age-related associations were significant.

### 3.4. Associations with Oral Care Attitudes

Spearman’s correlation analysis revealed no statistically significant associations between any of the personal background variables and attitudes toward oral care. Specifically, variables including gender (γ = 0.058, *p* = 0.386), age ranges (γ = 0.038, *p* = 0.570), years of nursing work experience (γ = 0.116, *p* = 0.082), job position (γ = −0.018, *p* = 0.787), educational level (γ = 0.070, *p* = 0.298), and previous professional oral care training hours (γ = 0.088, *p* = 0.186) all showed non-significant correlations.

Similarly, none of the sources of oral care knowledge were significantly correlated with attitudes. These included life experience (γ = 0.056, *p* = 0.400), self-study (γ = 0.118, *p* = 0.077), peer social network (γ = 0.077, *p* = 0.251), onboard training (γ = 0.018, *p* = 0.784), course content from professional training (γ = 0.121, *p* = 0.070), and supervisor guidance (γ = 0.045, *p* = 0.505).

One-way ANOVA further confirmed no significant differences in attitude scores by years of work experience (F(4, 220) = 2.603, *p* = 0.37) or educational level (F(4, 220) = 1.315, *p* = 0.265). However, attitudes differed significantly by age group (F(4, 220) = 3.487, *p* = 0.009), with post hoc analysis indicating that the 18–20 age group was positively associated with attitude scores (Spearman’s γ = 0.167, *p* = 0.012; see Table 4 for age-group correlations). Regarding educational level, secondary school education was positively associated with attitude scores (γ = 0.159, *p* = 0.017), whereas university education showed a negative association (γ = −0.147, *p* = 0.028). Primary and vocational education were not significantly associated.

### 3.5. Associations with Oral Care Practices

Spearman’s correlation analysis (Table 5) revealed that gender (r=0.135,p=0.043) and educational level (r=0.165,p=0.013) were positively associated with oral care practices. Additionally, reporting “professional training” as a source of oral care knowledge was positively associated with practices (γ = 0.170, *p* = 0.011).

### 3.6. Summary of Bivariate Findings

Bivariate analyses identified significant associations between select demographic variables and KAP domains. Gender was consistently associated with knowledge and practices. Age group differences were observed for knowledge and attitudes but not for practices. Educational level showed mixed associations: secondary education was positively associated with attitudes, while university education was negatively associated with knowledge and attitudes. Professional training as a knowledge source was positively associated with practices. Detailed multivariate results are presented in Section 3.7.

### 3.7. Multivariate Regression Analyses

Three separate stepwise multivariate regression models were constructed to identify significant predictors of KAP.

Predictors of oral care knowledge: University education (B = −1.240, SE = 0.416, β = −0.191, t = −2.977, *p* = 0.003), age 18–20 (B = −3.266, SE = 1.273, β = −0.168, t = −2.566, *p* = 0.011), and age 31–40 (B = −0.741, SE = 0.301, β = −0.158, t = −2.459, *p* = 0.015) were significantly associated with knowledge. The model explained 7.9% of the variance (adjusted R^2^ = 0.079, F(3, 221) = 7.417, *p* < 0.001).

Predictors of oral care attitudes: Age 18–20 (B = 10.325, SE = 3.219, β = 0.206, t = 3.208, *p* = 0.002), female gender (B = 4.033, SE = 1.437, β = 0.181, t = 2.806, *p* = 0.005), and primary education level (B = 5.658, SE = 2.794, β = 0.130, t = 2.025, *p* = 0.044) were significantly associated with attitudes. The model explained 7.5% of the variance (adjusted R^2^ = 0.075, F(3, 221) = 7.033, *p* < 0.001).

Predictors of oral care practices: Receiving oral care knowledge from a professional training programme (B = 2.205, SE = 0.801, β = 0.179, t = 2.754, *p* = 0.006), female gender (B = 3.636, SE = 1.499, β = 0.158, t = 2.425, *p* = 0.016), and secondary education level (B = 2.205, SE = 0.801, β = 0.179, t = 2.754, *p* = 0.006) were significantly associated with practices. The model explained 6.3% of the variance (adjusted R^2^ = 0.063, F(2, 222) = 6.937, *p* < 0.001). Table 6 shows stepwise multivariate regression predictors of KAP.

## 4. Discussion

This study examined the KAP regarding oral care among HAs and PCWs in Macao’s residential care homes for older adults. The findings revealed significant positive correlations among all three KAP domains, consistent with the theoretical premise that knowledge shapes attitudes, which in turn influence practices. However, the modest effect sizes (γ = 0.34–0.37) suggest that other factors, such as organizational support, time constraints, or resident cooperation, also play important roles.

### 4.1. Associations Among KAP

Consistent with prior research [21,22,23], knowledge was positively correlated with both attitudes and practices, and attitudes were positively correlated with practices. This pattern supports the KAP framework’s utility in geriatric oral care. Notably, subgroup analysis showed that among HAs, knowledge was not significantly associated with practice, a finding also reported in intensive care unit settings [24,25]. This may reflect that HAs, despite higher knowledge, face systemic barriers (e.g., higher workload or responsibility for more dependent residents) that weaken the knowledge–practice relationship. This pattern of dissociation between knowledge and practice among more senior staff has been observed in other long-term care settings internationally, including Hong Kong [14] and Sweden [25], suggesting a systemic rather than context-specific phenomenon. The consistency of this finding across different healthcare systems strengthens the argument that interventions must move beyond knowledge transfer to address organizational and environmental barriers.

### 4.2. Factors Associated with Oral Care Knowledge

Only gender showed a statistically significant association with knowledge scores (*p* = 0.002), with HAs scoring higher than PCWs. This aligns with previous studies indicating that senior caregiving roles are associated with greater oral care knowledge [26]. However, this finding must be interpreted with extreme caution given the small male sample. Age group differences in knowledge were observed in bivariate analysis (*p* = 0.011) but were not consistent across regression models, and the negative association for the 31–40 age group was small (β = −0.158). University education was unexpectedly negatively associated with knowledge (β = −0.191). Given the small proportion of university-educated participants (14.2%) and the lack of a dose–response relationship with educational level, this finding should be interpreted cautiously and may reflect differences in prior training content rather than educational attainment per se.

A notable finding is that previous professional oral care training hours were not significantly associated with knowledge scores (*p* = 0.97). This contrasts with intervention studies showing that structured training improves knowledge [27]. The discrepancy may reflect the nature of training received in Macao’s residential facilities, mostly informal induction-based orientation rather than standardized, repeatable, competency-assessed programmes. This finding underscores the need for formalized, ongoing training with verified learning outcomes rather than one-time orientation sessions.

### 4.3. Factors Associated with Oral Care Attitudes

Attitudes differed significantly by age group (*p* = 0.009), with the 18–20 age group showing a positive association (Spearman’s γ = 0.167, *p* = 0.012). However, this subgroup comprised only three participants (1.3%), making this finding potentially unreliable and not generalizable. Therefore, no firm conclusions or policy recommendations should be drawn from this subgroup.

The finding that university education was negatively associated with attitudes (γ = −0.147, *p* = 0.028) while primary education showed a positive association (B = 5.658, *p* = 0.044) is counterintuitive. One possible explanation is that individuals with higher formal education may have more critical attitudes toward workplace conditions or resource limitations, which could manifest as lower attitude scores on standardized measures. Alternatively, this finding may be specific to Macao’s context, where university-educated HAs reported receiving oral care knowledge primarily from professional training programmes (61.9%) compared with PCWs, potentially leading to higher expectations that are not met by workplace realities. This pattern warrants further qualitative investigation.

### 4.4. Factors Associated with Oral Care Practices

Professional training as a knowledge source was positively associated with practice scores (γ = 0.170, *p* = 0.011), suggesting that formal education content, as opposed to informal sources such as peer teaching or life experience, may be more effective in translating knowledge into action. Female gender and secondary education were also positively associated with practices. The association with secondary education, but not university education, may indicate that practical, hands-on training at a technical level is more directly applicable to daily oral care tasks than theoretical knowledge acquired at a higher educational level.

Better oral care practices were associated with female gender, higher educational level, and reporting “course content from professional training” as a knowledge source. These findings are partially consistent with previous research [27] but contrast with a past study that identified “self-learning” as a key predictor [25,27]. The difference may reflect regional variations in training curricula: many PCWs in Macao receive foundational clinical skills training in vocational or junior college programs, which may emphasize practical oral care procedures.

Notably, neither age nor years of work experience was significantly associated with practice scores. This diverges from studies suggesting that older or more experienced staff demonstrate better compliance [27,28], but aligns with evidence that younger staff may use online resources to access evidence-based practices [29,30]. In the resource-constrained, task-heavy environment of nursing homes, practice quality may depend more on institutional support and training accessibility than on age or tenure alone.

### 4.5. Practical Implications

The positive intercorrelations among KAP domains suggest that improving knowledge through targeted training could yield concurrent benefits for attitudes and practices. Specifically, training should address identified knowledge gaps, such as denture care and misconceptions about age-related tooth loss. Given that most participants received oral care knowledge through on-the-job training and peer communication, structured, repeatable training modules, potentially delivered by outreach dental professionals, could be effective.

Regarding gender findings, although gender was associated with knowledge and practices in bivariate analyses and female gender predicted attitudes in multivariate regression (adjusted R^2^ = 7.5%), these findings must not be used to develop gender-based policies or interventions. Three considerations support this caution: (1) the very small number of male participants (*n* = 16, 7.1%) means that gender-related findings are statistically unstable and may reflect sampling artifacts rather than true population differences; (2) the explained variance for the attitudes model was only 7.5%, indicating that gender accounts for a small portion of overall variation; and (3) there is no theoretical or clinical rationale for tailoring oral care training by gender. Future studies with larger and more balanced samples are needed before any gender-specific recommendations can be considered. 

### 4.6. Contribution to International Literature

This study makes several contributions beyond its local descriptive value. First, it is one of the few KAP studies to focus specifically on HAs and PCWs, the frontline staff who perform most daily oral care, rather than registered nurses, who are the typical focus of international research [13,14]. Second, by applying the COM-B model [18], the study demonstrates how capability (knowledge), opportunity (workplace resources), and motivation (attitudes) interact to influence behavior, offering a framework that can be adapted in other long-term care settings globally. Third, Macao’s unique context, rapid aging, Eastern–Western healthcare integration, and absence of outreach dental services, provides a natural experiment for other rapidly aging societies facing similar infrastructure gaps. The finding that most providers acquired knowledge through informal induction training and personal experience (64.9% and 62.7%, respectively) rather than structured professional development is consistent with studies from other Asian [14,18] and European [26] settings, suggesting that this is a widespread but modifiable problem.

### 4.7. Limitations

Several limitations warrant consideration. First, the cross-sectional design precludes causal inference. Second, findings are specific to Macao and may not generalize to other healthcare systems or cultural contexts without appropriate adaptation. However, as argued above, the barriers identified, lack of structured training, reliance on informal knowledge sources, and dissociation between knowledge and practice, are shared across many long-term care settings internationally, enhancing the transferability of key insights. Third, small subgroup sizes (e.g., age 18–20, *n* = 3; male providers, *n* = 16) mean that results involving these groups are statistically unstable and should not drive policy recommendations. Fourth, the relatively low adjusted R^2^ values for the regression models (7.9% for knowledge, 7.5% for attitudes, 6.3% for practices) indicate that demographic and training variables explain only a small portion of the variance in KAP outcomes. Unmeasured factors, including organizational culture, staffing ratios, availability of oral care supplies, leadership support, and resident cooperation, likely account for the remaining variance and should be prioritized in future research. Fifth, reliance on self-reported practices may introduce social desirability bias.

### 4.8. Future Research Direction

Future studies should employ longitudinal or observational designs to establish temporal relationships and directly observe practices rather than relying on self-report. Expanding to all elderly care facilities in Macao would improve representativeness. Qualitative methods (e.g., semi-structured interviews or focus groups) are needed to understand why knowledge does not consistently translate into practice among HAs and why university education was negatively associated with attitudes. Intervention studies testing structured, repeatable training programmes with objective competency assessment are a priority. Cross-national comparative studies involving Macao and other Asian jurisdictions (e.g., Hong Kong, Singapore, mainland China) would help distinguish context-specific from universal barriers to oral care in institutionalized aging populations.

## 5. Conclusions

In this cross-sectional study, oral care KAP of HAs and PCWs in Macao’s residential care homes were positively but modestly correlated. Most providers acquired oral care knowledge through induction training and personal experience. These findings highlight the need for structured, repeatable training with verified competency outcomes and suggest that Macao’s data can contribute to international efforts to improve oral care in institutionalized aging populations. Caution is warranted in interpreting gender- and age-related findings due to small subgroup sizes. Future study should employ longitudinal or observational designs and include all elderly care facilities in Macao.

## Figures and Tables

**Table 1:** Participants’ demographic characteristics (N =225).

	Overall	PCW	HA	Difference
	N (%)	N (%)	N (%)	*p*
Gender				0.003 **
Male Female	16 (7.1)	11 (4.9)	5 (2.2)	
	209 (92.9)	143 (63.6)	66 (29.3)	
Age Range (years)				0.019
18 to 20 21 to 30 31 to 40 41 to 50 51 or older	3 (1.3)	1 (0.4)	2 (0.9)	
	49 (21.8)	7 (3.1)	42 (18.7)	
	84 (37.3)	63 (28.0)	21 (9.3)	
	85 (37.8)	80 (35.6)	5 (2.2)	
	4 (1.8)	3 (1.3)	1 (0.5)	
Years of nursing work experience (Years)				0.115
Less than 1	18 (8.0)	11 (4.9)	7 (3.1)	
1 to 3	59 (26.2)	48 (21.3)	11 (4.8)	
3 to 5	63 (28.0)	52 (23.1)	11 (4.8)	
5 to 10	63 (28.0)	40 (17.8)	23 (10.2)	
10 years and more	22 (9.8)	3 (1.3)	19 (8.5)	
Work position				0.549
Personal Care Workers (PCW)	154 (68.4)	-	-	
Health Assistants (HA)	71 (31.6)	-	-	
Educational Levels				0.207
Primary School	4 (1.8)	4 (1.8)	0	
Secondary School	89 (39.6)	89 (39.6)	0	
Vocational School	57 (25.3)	49 (21.8)	8 (3.6)	
Junior college	43 (19.1)	10 (4.4)	33 (14.7)	
University (bachelor’s degree)	32 (14.2)	2 (0.9)	30 (13.3)	
Previously received professional oral care training hours				0.730
None	97 (43.1)	72 (0.3)	25 (11.1)	
Two hours or less	83 (36.9)	61 (27.1)	22 (0.98)	
Two to four hours or less	26 (11.6)	11 (4.8)	15 (6.7)	
Four to eight hours or less	7 (3.1)	5 (2.2)	2 (0.9)	
Eight to twelve hours or less	1 (0.4)	1 (0.4)	0	
Twelve hours or more	11 (4.9)	4 (1.8)	7 (3.1)	
Sources of oral care knowledge †				0.081
Life Experience	141 (62.7)	100 (44.4)	41 (18.3)	
Self-Study	75 (33.3)	46 (20.4)	29 (12.9)	
Word of mouth teaching among peers	96 (42.7)	63 (28.0)	33 (14.7)	
Onboard Training	143 (63.6)	102 (45.3)	41 (18.2)	
Course Content from Professional Learning	82 (36.5)	38 (16.9)	44 (19.6)	
Supervisor Guidance	109 (48.4)	86 (38.2)	23 (10.2)	

† Participants could select multiple sources; percentages do not sum to 100%. ** *p* < 0.01.

**Table 2:** Correlations among KAP (*n* = 225) (Spearman’s γ).

	Knowledge	Attitudes	Practices
Knowledge			
Spearman’s γ		0.34	0.35
*p*		<0.001 ***	<0.001 ***
Attitudes			
Spearman’s γ	0.34		0.37
*p*	<0.001 ***		<0.001 ***
Practices			
Spearman’s γ	0.35	0.37	
*p*	<0.001 ***	<0.001 ***	

*** *p* < 0.001.

**Table 3:** Chi-square analysis of personal background variables and knowledge.

	Value	df	*p*
Gender	33.27	13	0.002 **
Job Position	18.602	13	0.14
Educational Level	55.47	52	0.35
Previously received professional oral care training hours	45.98	65	0.97
Years of nursing work experience	55.42	52	0.35
Sources of Knowledge			
Life Experience	12.60	13	0.48
Self-Study	12.826	13	0.46
Word of mouth teaching among peers	14.096	13	0.37
Onboard Training	11.20	13	0.59
Professional Learning	14.51	13	0.34
Supervisor Guidance	13.708	13	0.39

** *p* < 0.001; Note: Degrees of freedom (df) reflect the number of categories within each variable after accounting for missing data and variable recoding. For Educational Level, the variable included six categories (Primary School, Secondary School, Vocational School, Junior College, University, and missing responses), yielding df = (6 − 1) × (2 − 1) = 5 for the 2 × 6 chi-square analysis. Some df values appear high due to the inclusion of multiple response categories and stratification of continuous variables (e.g., knowledge scores categorized into quartiles).

**Table 4:** Correlations between age groups and attitudes (Spearman’s γ).

Age Groups (Years)	Attitudes	
	Spearman’s Correlation	*p*
18 to 20	0.167	0.012 *
21 to 30	0.081	0.227
31 to 40	−0.143	0.032
41 to 50	0.022	0.739
51 years	0.044	0.508

* *p* < 0.05.

**Table 5:** Analysis of personal background variables and oral care practices (Spearman’s γ).

	Practices	
	Spearman’s Correlation	*p*
Gender	0.135	0.043 *
Age ranges	−0.122	0.068
Years of nursing work experience	0.034	0.613
Job positions	0.081	0.228
Educational levels	0.165	0.013 *
Previous professional oral care training (hours)	0.064	0.336
Sources of knowledge		
Life experience	0.080	0.231
Self-Study	0.131	0.050
Peer social network	0.049	0.465
Onboard training	−0.034	0.613
Professional training	0.170	0.011 *
Supervisor guidance	0.004	0.951

* *p* < 0.05.

**Table 6:** Stepwise multivariate regression predictors of KAP.

Outcome	Predictor	B (SE)	β	t	*p*
Knowledge (Adjusted R^2^ = 0.079)	University education	−1.240 (0.416)	−0.191	−2.977	0.003
	Age 18–20	−3.266 (1.273)	−0.168	−2.566	0.011
	Age 31–40	−0.741 (0.301)	−0.158	−2.459	0.015
Attitudes (Adjusted R^2^ = 0.075)	Age 18–20	10.325 (3.219)	0.206	3.208	0.002
	Female gender	4.033 (1.437)	0.181	2.806	0.005
	Primary education	5.658 (2.794)	0.130	2.025	0.044
Practices (Adjusted R^2^ = 0.063)	Professional training	2.205 (0.801)	0.179	2.754	0.006
	Female gender	3.636 (1.499)	0.158	2.425	0.016
	Secondary education	2.205 (0.801)	0.179	2.754	0.006

All models *p* ≤ 0.005.

## Data Availability

The data presented in this study are available on request from the corresponding author. The data are not publicly available for confidentiality purposes.

## Supplementary Materials

The following supporting information can be downloaded at: https://www.mdpi.com/article/10.3390/geriatrics11030058/s1. File S1: The Strengthening the Reporting of Observational Studies in Epidemiology (STROBE) Statement.

## Abbreviations

The following abbreviations are used in this manuscript:

COM-B	Capability, Opportunity, Motivation-Behaviour
HA	Health Assistant
KAP	Knowledge, Attitudes, Practices
PCW	Personal Care Worker
STROBE	Strengthening the Reporting of Observational Studies in Epidemiology
