# Supplementary material for: Knowledge, Attitudes, and Practices of Oral Care for Residential Older People Among Healthcare Providers in Macao
Source: Geriatrics (Basel). 2026 May 13;11(3):58. doi: 10.3390/geriatrics11030058 (PMC13214962; doi:10.3390/geriatrics11030058)
Supplement: Supplementary file 1 [file geriatrics-11-00058-s001.zip › geriatrics-4272778-supplementary.pdf]

STROBE Statement—Checklist of items that should be included in reports of ***cross-sectional studies***

	Item No	Recommendation	Page No
**Title and abstract**	1	(*a*) Indicate the study's design with a commonly used term in the title or the abstract	Page 1
		(*b*) Provide in the abstract an informative and balanced summary of what was done and what was found	Page 1
**Introduction**			
Background/rationale	2	Explain the scientific background and rationale for the investigation being reported	Pages 1-2
Objectives	3	State specific objectives, including any prespecified hypotheses	Page 2
**Methods**			
Study design	4	Present key elements of study design early in the paper	Page 2-3
Setting	5	Describe the setting, locations, and relevant dates, including periods of recruitment, exposure, follow-up, and data collection	Page 3
Participants	6	(*a*) Give the eligibility criteria, and the sources and methods of selection of participants	Page 3
Variables	7	Clearly define all outcomes, exposures, predictors, potential confounders, and effect modifiers. Give diagnostic criteria, if applicable	Pages 3-4
Data sources/ measurement	8*	For each variable of interest, give sources of data and details of methods of assessment (measurement). Describe comparability of assessment methods if there is more than one group	Pages 3-4
Bias	9	Describe any efforts to address potential sources of bias	NA
Study size	10	Explain how the study size was arrived at	Page 3
Quantitative variables	11	Explain how quantitative variables were handled in the analyses. If applicable, describe which groupings were chosen and why	Pages 3-4
Statistical methods	12	(*a*) Describe all statistical methods, including those used to control for confounding	Pages 5
		(*b*) Describe any methods used to examine subgroups and interactions	Pages 5
		(*c*) Explain how missing data were addressed	NA
		(*d*) If applicable, describe analytical methods taking account of sampling strategy	Pages 5
		(*e*) Describe any sensitivity analyses	NA
**Results**			
Participants	13*	(a) Report numbers of individuals at each stage of study—eg numbers potentially eligible, examined for eligibility, confirmed eligible, included in the study, completing follow-up, and analysed	Pages 5-6
		(b) Give reasons for non-participation at each stage	Not applicable
		(c) Consider use of a flow diagram	Nil
Descriptive data	14*	(a) Give characteristics of study participants (eg demographic, clinical, social) and information on exposures and potential confounders	Page 6-10
		(b) Indicate number of participants with missing data for each variable of interest	Page 6-10

Outcome data	15*	Report numbers of outcome events or summary measures	Pages 6-10
Main results	16	(*a*) Give unadjusted estimates and, if applicable, confounder-adjusted estimates and their precision (eg, 95% confidence interval). Make clear which confounders were adjusted for and why they were included	Pages 6-10
		(*b*) Report category boundaries when continuous variables were categorized	Pages 6-10
		(*c*) If relevant, consider translating estimates of relative risk into absolute risk for a meaningful time period	Not applicable
Other analyses	17	Report other analyses done—eg analyses of subgroups and interactions, and sensitivity analyses	Not applicable
**Discussion**			
Key results	18	Summarise key results with reference to study objectives	Pages 10-11
Limitations	19	Discuss limitations of the study, taking into account sources of potential bias or imprecision. Discuss both direction and magnitude of any potential bias	Pages 11-12
Interpretation	20	Give a cautious overall interpretation of results considering objectives, limitations, multiplicity of analyses, results from similar studies, and other relevant evidence	Pages 10-12
Generalisability	21	Discuss the generalisability (external validity) of the study results	Page 11-12
**Other information**			
Funding	22	Give the source of funding and the role of the funders for the present study and, if applicable, for the original study on which the present article is based	Page 12

*Give information separately for exposed and unexposed groups.

Note: An Explanation and Elaboration article discusses each checklist item and gives methodological background and published examples of transparent reporting. The STROBE checklist is best used in conjunction with this article (freely available on the Web sites of PLoS Medicine at http://www.plosmedicine.org/, Annals of Internal Medicine at http://www.annals.org/, and Epidemiology at http://www.epidem.com/). Information on the STROBE Initiative is available at www.strobe-statement.org.
